# Integrated metabolome and transcriptome analyses reveal the response mechanism under drought stress of *Callicarpa bodinieri* ‘JinYe'

**DOI:** 10.3389/fpls.2026.1714253

**Published:** 2026-03-26

**Authors:** Youju Ye, Qingdi Hu, Xule Zhang, Xiaohua Ma, Renjuan Qian, Jian Zheng

**Affiliations:** Zhejiang Institute of Subtropical Crops, Wenzhou Key Laboratory of Resource Plant Innovation and Utilization, Wenzhou, Zhejiang, China

**Keywords:** *Callicarpa bodinieri* ‘JinYe’, drought stress, integrated analysis, metabolism, transcriptome

## Abstract

‘JinYe’ is a different cultivar of *Callicarpa bodinieri* whose leaves are golden yellow. Drought stress occurs in many regions and has a significant effect on plant development. In this study, the metabolome and transcriptome of *C. bodinieri* ‘JinYe’ were analyzed to elucidate its response mechanism to drought stress, after which differentially expressed genes and metabolites were identified. Integrated analysis of the metabolome and transcriptome revealed that the lipid pathway and *Cluster-145547.39844* (*DNA-binding with one finger, or the DOF* gene) may play significant roles in the response to drought stress in *C. bodinieri* ‘JinYe’. These results provide a basis for exploring the response mechanism of *C. bodinieri* ‘JinYe’ to drought stress.

## 1 Introduction

*Callicarpa bodinieri* belongs to *Callicarpa* L (Markovi et al., 2018)*. C. bodinieri* is a deciduous shrub (usually approximately 2 meters tall) whose leaf blade has an ovate-oblong to elliptic shape, apex is long acuminate to mucronate, base is cuneate, margin is serrate, and is abaxially grey–brown (Ma et al., 2011). The corolla of *C. bodinieri* is purple, with an ellipse-shaped anther, and the fruit is spherical, flowering in June–July and bearing fruit in August–November (Lin et al., 2011). The root of *C. bodinieri* can be used in medicine to treat irregular menstruation, virtual fatigue, leucorrhea, postpartum gas pain, cold wind cold, and sesame oil can be used externally to cure erysipelas and treat disturbances in the menstrual cycle and blood flow (Jones et al., 2007). *C. bodinieri* is distributed in most areas of China and is usually found in forest margins and thickets (200–2300 m) (Yamasaki et al., 2007). *C. bodinieri* ‘JinYe’ was the variety selected for our *C. bodinieri* cultivation process. This variety is the offspring of *C. bodinieri* and harbors a new mutation that causes its leaves to be golden yellow in color. ‘JinYe’ is a deciduous shrub whose golden yellow leaves last for approximately 60 d; the mature leaves turn yellow–green, and the best ornamental period is March to June.

Among the various stress factors, drought stress occurs in almost all regions and has a significant effect on agricultural land, leading to the disruption of physiological, biochemical and molecular processes, and persistent drought stress affects plant growth (Raza et al., 2025; Ahmad et al., 2025; Rezk et al., 2025). Notably, drought stress results in insufficient water supply and subsequently influences development in plants (Shanker et al., 2014; Wang et al., 2023; Zhang et al., 2024). The impact of drought stress on plant development varies on the basis of various factors, such as developmental stage, intensity, duration and intrinsic plant characteristics, involving complex and subtle signal sensing and transduction mechanisms (Zhu, 2002; Shoukat et al., 2025). Understanding the mechanism underlying the response to drought stress is a prerequisite for further stress tolerance studies in plants (Ali et al., 2017; Zuo et al., 2024).

Abscisic acid (ABA) plays a vital role in the response to drought stress in plants and is referred to as a “growth-inhibiting plant hormone” (Bharath et al., 2021). In addition, ABA is an essential hormone for plants throughout their life cycle, and the levels of ABA increase rapidly in plants under drought stress (Cao et al., 2025). Under drought conditions, ABA activates SnRK2.6, further phosphorylating it and inhibiting the activity of KAT1, preventing potassium ions from entering guard cells and eventually leading to stomatal closure (Lim et al., 2015). Additionally, the functional quality of strawberry fruits is regulated through an ABA-dependent mechanism (Perin et al., 2019). In soybean, the ABA content increases during key flower developmental stages in response to drought stress (Liu et al., 2003). In *Zea mays*, ABA participates in regulating the response to drought stress (Zhang et al., 2012).

Furthermore, multiple transcription factor families, such as the *DOF(DNA-binding with one finger)* family, *bHLH (basic helix-loop-helix)* family, *WRKY* family, *MYB (v-myb avian myeloblastosis viral oncogene homolog)* family and *bZIP (basic leucine zipper)* family, have been shown to be involved in the plant response to drought stress (Hu et al., 2025; Joshi et al., 2016; Manna et al., 2021; Song et al., 2024; Zheng et al., 2025). Under drought stress, these transcription factors participate in the ABA signaling pathway and subsequently form a complex regulatory network (Hussain et al., 2021; Spinelli et al., 2024). *AtbHLH68* is involved in ABA homeostasis in the response of *Arabidopsis thaliana* to drought stress (Le et al., 2017). In *Zea may*, *Zmhdz9* regulates ABA accumulation to promote resistance to drought stress (Jiao et al., 2024).

In this study, we explored the molecular mechanism underlying the response of *C. bodinieri* ‘JinYe’ to drought stress. First, physiological property measurements and hormone detection were conducted, and then, transcriptome sequencing and primary metabolome sequencing were performed on the samples from plants grown under drought conditions to select key genes and metabolites. RT–qPCR was conducted to verify key genes, and integrated analysis between key metabolites and genes was performed.

## 2 Materials and methods

### 2.1 Plant materials and drought stress treatment

*C. bodinieri* ‘JinYe’ plants were cultivated at the Zhejiang Institute of Subtropical Crops (120°63’54”E, 27°99’88”N), Wenzhou, Zhejiang Province, China. All the plants were maintained under stable conditions in June 2024, with a daytime (16 h) temperature of 35 °C and a nighttime (8 h) temperature of 25 °C. The initial soil moisture was 75%. Drought stress was imposed by withholding irrigation, and three gradient levels were established, namely, normal irrigation (no withholding irrigation) (D0), 5-day withholding irrigation (D5), and 10-day withholding irrigation (D10). Three samples in each treatment group were collected for subsequent research. Thus, a total of 9 samples were collected and then stored at -80 °C after liquid nitrogen treatment.

### 2.2 Physiological property measurement and hormone detection

The leaf weight, dry weight, length, and width of 9 samples (D0, D5, and D10; three samples in each group) were measured using an electronic balance and a Vernier calliper (3 replicates per treatment). We calculated the pot weight and fresh weight to obtain the leaf moisture content data for each sample. High-performance liquid chromatography (HPLC) was used for ABA content detection. The detailed protocol is as follows. First, ABA extraction and purification were conducted following the protocol of Li et al. (2016). The samples were homogenized on ice and extracted in the dark for 15 hours. The supernatant was removed after centrifugation (5000 g/10 min), which was repeated twice, and the pellet was then dried under a vacuum and dissolved in ammonium acetate. Afterwards, the supernatant was added to a solution with PVPP and DEAE-Sephadex G-25 (Whatman, Maidstone, UK) and filtered through a Chromosep C18 column (C18Sep-Pakcartridge, Waters, Milford, MA, USA) for purification (Cai et al., 2014).

### 2.3 Transcriptome sequencing and data analysis

Total RNA from 9 sample (D0, D5 and D10, three repeats) leaves was extracted following a standard protocol (Tiangen, Beijing, China) and then reverse transcribed into cDNA to obtain a library (Hansen et al., 2010; Cock et al., 2010). The raw data included a small number of reads with sequencing connectors or with low sequencing quality (Altschul et al., 1997; Grabherr et al., 2011). The read counts were mapped onto the transcriptome and assembled with PacBio (Qian et al., 2023). The DESeq2 R package was used to identify differentially expressed genes (DEGs) (Love et al., 2014). The GO and KEGG databases were used to annotate the unigenes (Kanehisa et al., 2008; Young et al., 2010).

### 2.4 Primary metabolome sequencing and data analysis

The GC–MS instrument system consisted of an Ultra Performance Liquid Chromatographer (SHIMADZU Nexera X2, https://www.shimadzu.com.cn/) and a Tandem Mass Spectrometer (Applied Biosystems 4500 QTRAP, http://www.appliedbiosystems.com.cn/). The liquid chromatography conditions were as follows: Chromatographic column: Agilent SB-C18 (1.8 µm, 2.1 mm × 100 mm); mobile phase: Phase A was ultrapure water (with 0.1% formic acid added), and Phase B was acetonitrile (with 0.1% formic acid added); elution gradient: The proportion of Phase B was 5% at 0.00 min, linearly increased to 95% within 9.00 min, maintained at 95% for 1 min, decreased to 5% from 10.00 to 11.10 min, and equilibrated at 5% until 14 min; flow rate: 0.35 mL/min; column temperature: 40 °C; and injection volume: 4 μL. The mass spectrometry conditions were as follows: electrospray ionization (ESI) temperature, 550 °C; ion spray voltage (IS), 5500 V (positive ion mode)/4500 V (negative ion mode); and ion source gas I (GSI), gas II (GSII), and curtain gas (CUR), which were set to 50, 60, and 25 psi, respectively; and the collision-induced dissociation parameters were set to high. Instrument tuning and mass calibration were performed with 10 μmol/L and 100 μmol/L polypropylene glycol solutions in QQQ and LIT modes, respectively. QQQ scanning was performed in MRM mode, with the collision gas (nitrogen) set to medium. The declustering potential (DP) and collision energy (CE) for each MRM ion pair were optimized through further adjustments. A specific set of MRM ion pairs was monitored during each period according to the metabolites eluted during that period. Sixty milligrams of each sample (9 sample) was extracted and then stored at -80 °C for 2 min. After centrifugation (60 Hz/2 min), the samples were ultrasonicated (25 °C/30 min). Afterwards, 200 μL of chloroform was added and vortexed (1 min), followed by the addition of 400 μL of water and vortexing (1 min). After ultrasonication for 30 min at 25 °C and centrifugation for 10 min at 4 °C (12,000 rpm), 80 μL of 15 mg mL^-1^ methoxylamine hydrochloride in pyridine was mixed to obtain a mixture Eighty microliters of BSTFA (with 1% TMCS) and 20 μL of n-hexane were added, and the mixture was vortexed (25 °C/2 min) and then derivatized at 70 °C. The samples were subjected to GC–MS analysis after cooling (30 min) according to previous methods (Chen et al., 2013; Thévenot et al., 2015). Mass spectrometry data were processed using Analyst 1.6.3 software. ChromaTOF and ChemStation software were used for data processing, while for annotated metabolites, the National Institute of Standards and Technology (NIST) database was used. The OmicStudio tool (https://www.omicstudio.cn/tool) was used for integrated analysis (Lv et al., 2023). Pearson correlation was used to analyze correlations. DEMs with a p value <0.05 were identified. The p value was calculated with the DEG algorithm in the R package for experiments with biological replicates (Anders, 2010).

### 2.5 RT–qPCR experiment

In the RT–qPCR experiments, the *actin* gene was used as the internal reference gene, and primers were designed through Oligo 7 (**Supplementary Table 1**). The reaction system used was described previously by Ye (Ye et al, 2024). To ensure the accuracy of the experiment, three technical replicates were conducted. The 2^−ΔΔ^ CT method was used to calculate relative expression.

### 2.6 Integrated analysis of key metabolites and differentially expressed genes

Lipid compounds between D0 and D10 were the most abundant category of metabolites; thus, 11 lipid compounds (*P* < 0.05) were selected for further integrated analysis of the differentially expressed genes. A total of 8 genes (3 structural genes and 5 TF genes) were identified. Advanced Cor link was performed using the OmicStudio tools at https://www.omicstudio.cn/tool with the Pearson method.

## 3 Results

### 3.1 Leaf water content and ABA content of *C. bodinieri* ‘JinYe’ under drought stress

**Figure 1:**
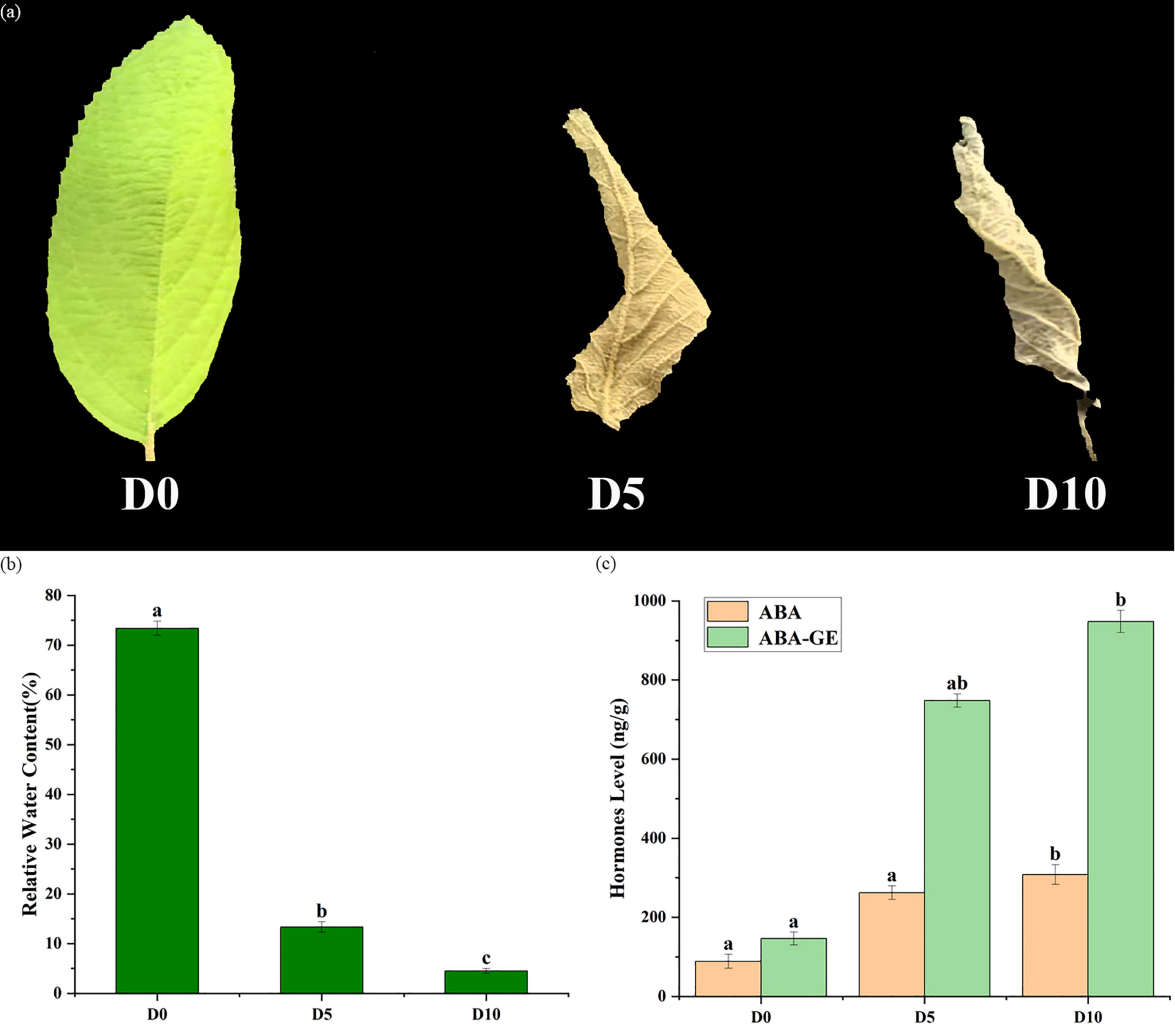
Leaf characteristics and water content of *C. bodinieri* under drought stress. **(a)** Untreated sample (D0) and drought-treated sample (D5 and D10); scale bar: 1 cm. **(b)** Leaf condition and water content of *C. bodinieri* under drought stress. Significant differences were determined through Student’s *t* test. Different lowercase letters indicate significant differences (P < 0.05). **(c)** ABA content variation in different drought treatment groups.

Leaf samples from the untreated and drought-treated plants are presented in **Figure 1a**. the leaves gradually withered under drought stress. As the duration of drought treatment increased, the degree of withering increases, and the leaves even begin to curl.

Moreover, the changes in the water content of *C. bodinieri* leaves under drought stress were recorded and are shown in **Figure 1b**. The results revealed that in the leaves of D0 (CK), the leaf water content reached 75%, while in the leaves of D5 and D10, the water content was 13% and 5%, respectively (**Figure 1b**). These data are also consistent with our observations of the leaves. The ABA content was analyzed under no-drought-stress conditions (D0), after 5 days of drought treatment (D5) and after 10 days of drought treatment (D10). The results indicated that the ABA content was lowest in the CK group, whereas after 10 days of drought treatment, the ABA content was the greatest (**Figure 1c**). Further analysis revealed that ABA and ABA-GE were present in the plants grown under drought stress conditions compared to the plants grown under normal conditions.

### 3.2 Transcriptome analysis of *C. bodinieri* ‘JinYe’ under different drought treatments

A total of 63.62 Gb of raw data and 62.88 Gb of clean data were obtained in the transcriptome analysis of the 9 leaf samples from *“JinYe”C. bodinieri* (**Supplementary Table 2**). After assembly, 444985 transcripts and 244362 unigenes were obtained.

**Figure 2:**
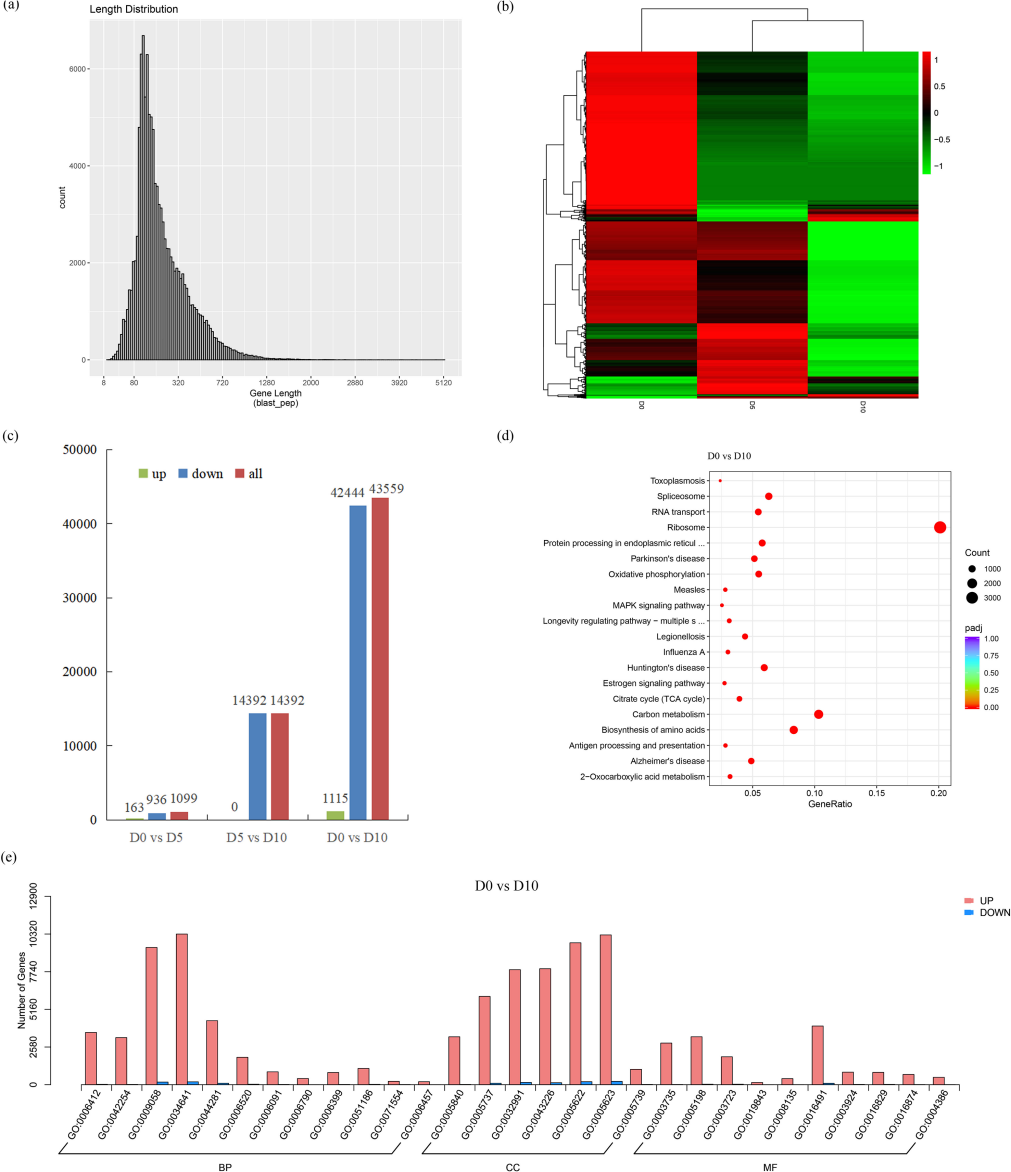
Transcriptome analysis *of “JinYe”C. bodinieri* under different drought treatments. **(a)** CDS length distribution. **(b)** Heatmap of unigenes. **(c)** DEGs in the 3 groups (D0 vs. D5, D5 vs. D10, D0 vs. D10). The green bar represents upregulated DEGs, while the blue bar represents downregulated DEGs; all DEGs are represented by a red color bar. The x-axis represents the comparison group, while the y-axis represents the number of DEGs. **(d)** KEGG enrichment analysis 0f D0 vs. D10. **(e)** GO enrichment analysis of D0 vs. D10.

On the basis of the transcriptome data, the DEGs were also analyzed in this study. The length distribution of the CDS indicated that the CDS length was mainly between 300 and 1200 bp (**Figure 2a**). The expression levels of the unigenes are presented in a heatmap (**Figure 2b**). A comparison of the drought-treated samples revealed 163 upregulated and 936 downregulated DEGs between D0 and D5 and 14392 downregulated DEGs between D5 and D10. D0 differed greatly from D10, with 42444 upregulated and 1115 downregulated DEGs between D0 and D10 (**Figure 2c**). KEGG analysis of D0 vs. D10 revealed that most genes were related to the ribosome, followed by carbon metabolism (**Figure 2d**). GO enrichment analysis between D0 and D10 revealed that most genes belonged to the biological process (BP) category (**Figure 2e**).

### 3.3 Overall characteristics of the metabolome results and differentially abundant metabolite analysis

**Figure 3:**
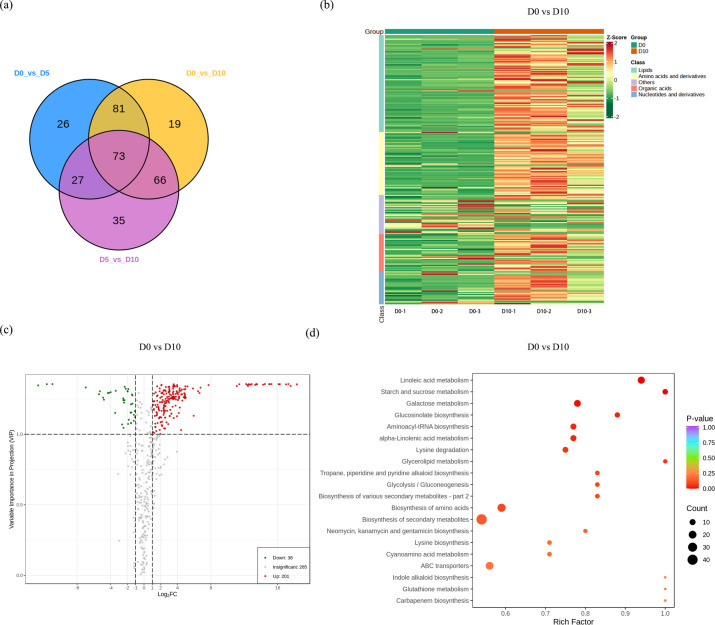
Differentially abundant metabolite analysis. **(a)** Differentially abundant metabolite Venn diagram. Different comparison groups are represented by different colored circles. **(b)** Heatmap of differentially abundant metabolite levels between D0 and D10. The green color represents a decrease, whereas the red color represents an increase. **(c)** Volcano plot of differentially abundant metabolites. **(d)** KEGG analysis of differentially abundant metabolites.

Primary metabolites were compared across the different drought treatment groups, and 504 differentially abundant metabolites were identified in this study (**Supplementary Figure 1a**). The characteristic ions of each substance were screened by triple quadrupole, and the signal strength (CPS) was obtained in the detector; finally, all the peak area integration results were generated (**Supplementary Figure 1b**). All 504 detected metabolites could be divided into 5 categories, including the subgroups of amino and derivatives (17.26%), lipids (31.94%), nucleotides and derivatives (12.5%), organic acids (20.63%) and others (17.66%) (**Supplementary Figure 1c**). Differentially abundant metabolites were also analyzed among the groups and are presented in a Venn diagram. A Venn diagram revealed the most differentially abundant metabolites in D0 vs. D10 (201 upregulated and 38 downregulated), followed by D0 vs. D5 (165 upregulated and 42 downregulated) and D5 vs. D10 (132 upregulated and 69 downregulated) (**Figure 3a**). Therefore, we focused our research on differentially abundant metabolites between D0 and D10. Subsequent content level and KEGG enrichment analyses between D0 and D10 were performed. A heatmap indicated that these differentially abundant metabolites of D0 and D10 mainly showed opposite expression trends. Among these differentially abundant metabolites, lipid compounds (115) were the most abundant in these compared groups, followed by amino acids and derivatives (62) (**Figure 3b**). A volcano plot revealed 201 differentially abundant metabolites with increased abundance and 38 differentially abundant metabolites with decreased abundance between D0 and D10 (**Figure 3c**). The functions of the differentially abundant metabolites between D0 and D10 were further annotated, and the results revealed that the biosynthesis of secondary metabolites was the most enriched term, followed by the biosynthesis of amino acids and ABC transporters (**Figure 3d**).

### 3.4 Differential expression analysis of key DEGs

**Figure 4:**
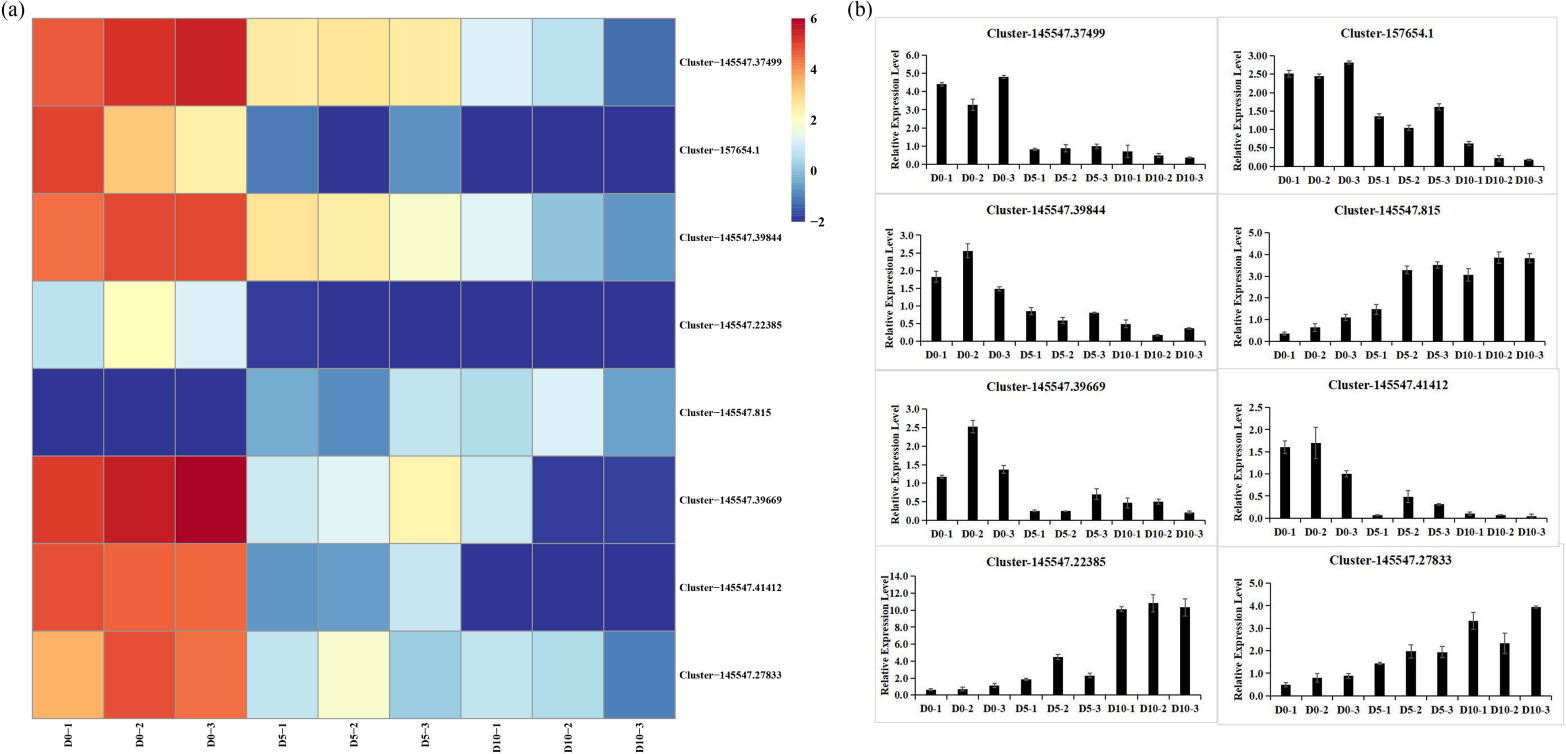
Identification of expression patterns of key DEGs. **(a)** Heatmap of key DEGs according to the FPKM values. **(b)** RT–qPCR results of key DEGs.

A total of 8 DEGs were selected for differential expression analysis by RT–qPCR: 2 *DOF* genes (*Cluster-145547.37499*, *Cluster-157654.1*, *and Cluster-145547.39844*), 1 *bZIP gene* (*Cluster-145547.22385*), 3 *MYB* genes (*Cluster-145547.815* and *Cluster-145547.39669* and *Cluster-145547.41412*), and 1 *bHLH* gene (*Cluster-145547.27833*). The RT–qPCR results were consistent with the FPKM results (**Figure 4**).

### 3.5 Integrated analysis of the transcriptome and metabolome in *C. bodinieri* ‘JinYe’

**Figure 5:**
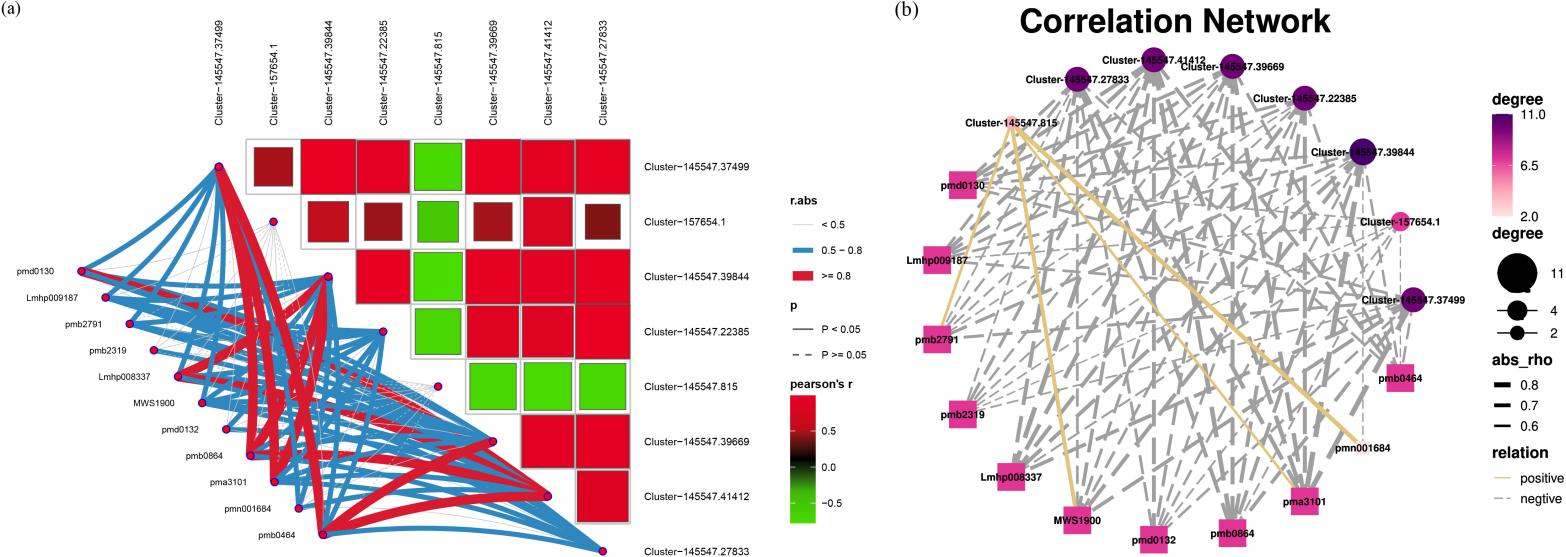
Integrated analysis between TF gene expression and lipid metabolite levels. **(a)** The correlation heatmap between gene expression and lipid metabolite levels. **(b)** The connection network between genes and lipid metabolites.

A metabolite–gene interaction network was constructed to further explore the molecular mechanisms underlying the response of *C. bodinieri* ‘JinYe’ to drought stress. Eleven differentially abundant metabolites whose P values were < 0.05 in all the compared groups were selected for the next study (**Supplementary Table 3**). To explore the effects of drought stress on lipid metabolism in *C. bodinieri*, we analyzed mainly the expression of genes that participate in lipid metabolism under drought stress; thus, 8 DEGs related to lipid metabolism were selected. The combined analysis of differentially abundant metabolites and DEGs revealed that most genes were negatively correlated with the levels of the metabolites, except Cluster-145547.815 (*MYB* gene). The expression of *Cluster-145547.39844* (*Dof* gene) exhibited the greatest correlation with the levels of these 11 lipid metabolites (**Figure 5**).

## 4 Discussion

### 4.1 Changes in ABA levels under drought stress

Climate change has become a major threat to global crop production, leading to various abiotic stresses, such as high temperatures, drought, and flooding. Therefore, to sustain stable growth and development under changing environmental conditions, an increasing number of plant drought resistance mechanisms have been elucidated (Wang et al., 2023). Plants exhibit drought resistance through multiple mechanisms, such as stomatal closure, cuticular wax synthesis in leaves, and increased root depth (Couvillion et al., 2023; Mu et al., 2025). Accumulating evidence indicates that hormones play important roles in stress responses (Lu et al., 2025; Yang et al., 2025).

As an important plant hormone, ABA regulates physiological and molecular processes. ABA activates the transcription of certain genes whose products of translation, such as enzymes that participate in the synthesis of osmotic agents, participate in the regulation of drought stress responses (Fujita et al., 2006). In tomato, inhibition of JA biosynthesis genes (AOC and OPR3) is relieved through the phosphorylation of CPK27 activated by ABA and ubiquitination degradation mediated by PUB22, promoting the accumulation of JA and ultimately improving drought tolerance (Zhu et al., 2025). We also reported that *PeFUS3* enhances drought resistance by upregulating *PePYL3* gene expression in the ABA signaling pathway (Liu et al., 2025). On the basis of these results, we speculate that ABA may participate in the response to drought stress in *C. bodinieri* ‘JinYe’ in this study.

### 4.2 Integrated analysis was performed to further explore the molecular mechanisms underlying the response of *C. bodinieri* ‘JinYe’ to drought stress

Transcriptome sequencing was performed in this study, and key DEGs were identified through RT–qPCR. The transcriptome results revealed the greatest number of DEGs (upregulated/downregulated) between the normal irrigation samples (D0) and the 10-day drought-treated samples (D10), regardless of whether the number of DEGs increased or decreased. Eight differentially expressed genes (DEGs) were demonstrated to potentially be involved in the response to drought stress through RT–qPCR.

To further explore the molecular mechanisms of *C. bodinieri* ‘JinYe’ under drought stress, 11 differential lipid metabolites were selected for correlation analysis with the DEGs in this study. The results indicated that *Cluster-145547.39844* (a *DOF* gene) is clearly involved in drought stress and was strongly correlated with the levels of lipid metabolites, which may play a vital role in the response to drought stress.

The *DOF* family is a plant-specific transcription factor family that possesses a highly conserved single Cys_2_/Cys_2_ zinc finger DNA-binding domain and is involved in multiple plant biological processes, including seed germination, flowering, leaf senescence, and stress responses (Zhang et al., 2022; Zou and Sun, 2023). In *A. thaliana*, the XND1-DOF4.6 interaction module negatively regulates water transport and drought resistance by modulating two pathways: xylem-mediated axial transport and aquaporin-mediated radial transport. On the one hand, DOF4.6 interacts with XND1 to increase the binding of XND1 to xylem-related target genes, thereby inhibiting xylem formation. On the other hand, DOF4.6 can directly repress the expression of aquaporins, thus impairing water transport and drought resistance (Ding et al., 2025). In rice, transcriptomic and metabolomic analyses of transgenic materials have demonstrated that *OsDof12* plays a novel positive regulatory role in drought tolerance mediated by the phenylpropanoid pathway (Shim et al., 2025). Wang et al. (2025) cloned the *IbDof2.1* gene in sweet potato and reported that its overexpression significantly increased drought resistance. On the basis of the results of this study, we hypothesize that *Cluster-145547.39844* (a *DOF* gene) may play an important role in the response of *C. bodinieri* ‘JinYe’ to drought stress.

## 5 Conclusion

In this study, metabolome and transcriptome analyses were performed to explore the response mechanism of *C. bodinieri* ‘JinYe’ to drought stress, after which differentially expressed genes and metabolites were identified. Correlation analysis of the metabolome and transcriptome revealed that lipid metabolites and *Cluster-145547.39844* (a *DOF* gene) may play a significant role in the response to drought stress in *C. bodinieri* ‘JinYe’. Our results provide a reference for understanding the response mechanism of *C. bodinieri* ‘JinYe’ to drought stress.

## Data Availability

The datasets presented in this study can be found in online repositories. The names of the repository/repositories and accession number(s) can be found below: https://www.ncbi.nlm.nih.gov/, PRJNA1314855.
