# Supplementary figures and images for: Integrated metabolome and transcriptome analyses reveal the response mechanism under drought stress of Callicarpa bodinieri ‘JinYe'
Source: Front Plant Sci. 2026 Mar 26;17:1714253. doi: 10.3389/fpls.2026.1714253 (PMC13061715; doi:10.3389/fpls.2026.1714253)

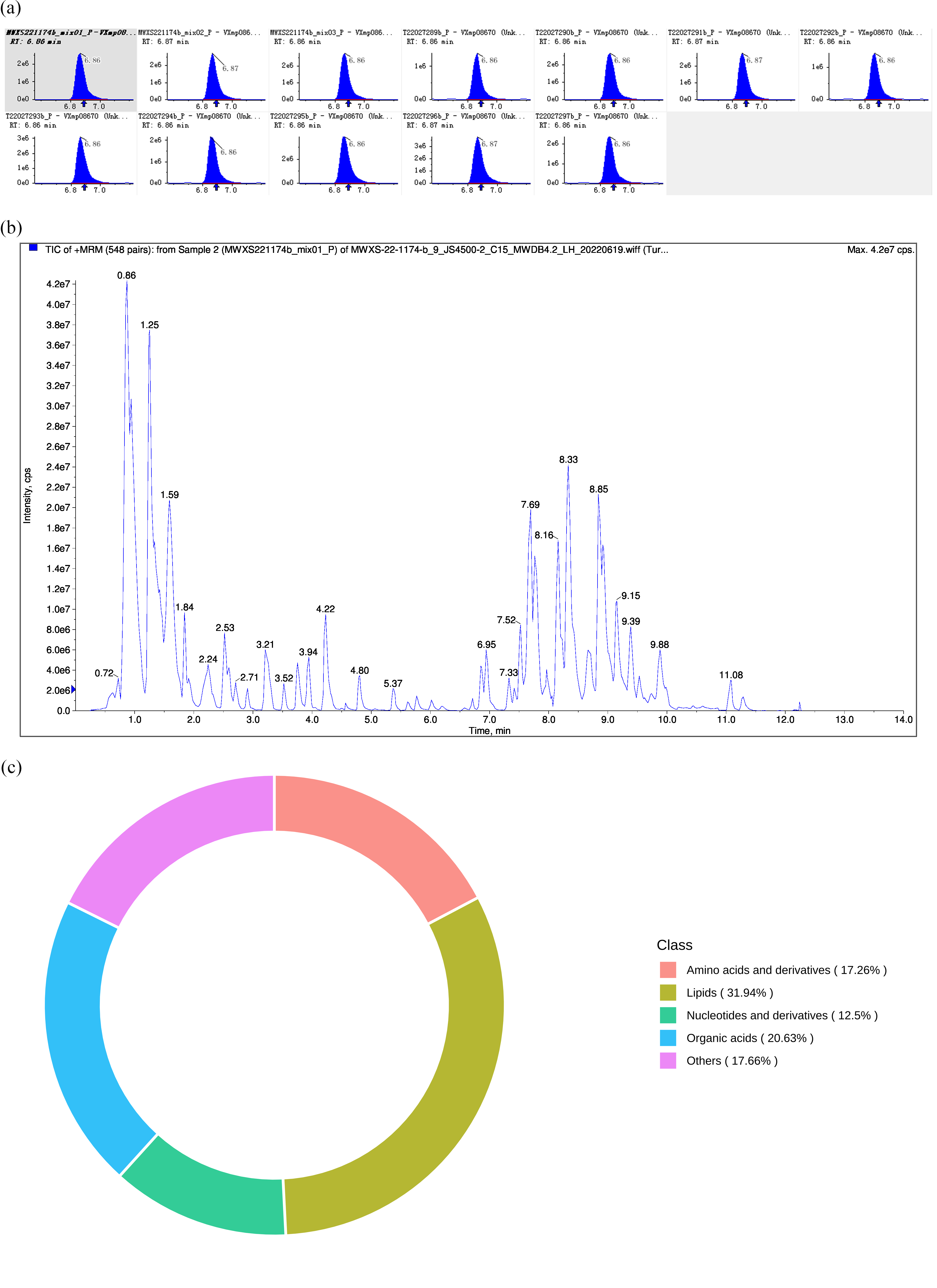

Supplement: Supplementary file 1 [file Image1.png]
